# Ocular effects of virtual reality headset wear in young adults

**DOI:** 10.1038/s41598-017-16320-6

**Published:** 2017-11-23

**Authors:** Philip R. K. Turnbull, John R. Phillips

**Affiliations:** 1 School of Optometry and Vision Science, The University of Auckland, Auckland, New Zealand; 2 Department of Optometry, Asia University, Taichung, Taiwan

## Abstract

Virtual Reality (VR) headsets create immersion by displaying images on screens placed very close to the eyes, which are viewed through high powered lenses. Here we investigate whether this viewing arrangement alters the binocular status of the eyes, and whether it is likely to provide a stimulus for myopia development. We compared binocular status after 40-minute trials in indoor and outdoor environments, in both real and virtual worlds. We also measured the change in thickness of the ocular choroid, to assess the likely presence of signals for ocular growth and myopia development. We found that changes in binocular posture at distance and near, gaze stability, amplitude of accommodation and stereopsis were not different after exposure to each of the 4 environments. Thus, we found no evidence that the VR optical arrangement had an adverse effect on the binocular status of the eyes in the short term. Choroidal thickness did not change after either real world trial, but there was a significant thickening (≈10 microns) after each VR trial (p < 0.001). The choroidal thickening which we observed suggest that a VR headset may not be a myopiagenic stimulus, despite the very close viewing distances involved.

## Introduction

The era of consumer-grade virtual reality (VR) is upon us, with a variety of inexpensive VR head mounted displays (HMDs) currently available. However, there is a perception that using VR HMDs may be associated with risks, possibly to the eyes, and several suppliers include non-specific warnings associated with HMD use. Here we investigate whether use of a VR HMD affects binocular vision status and whether there is evidence that it may be a stimulus for development of myopia (short-sightedness). VR HMDs create a sense of presence in a computer-generated world by presenting dichoptic images whose perspective shifts with the user’s head movements in the real world. Inside the HMD helmet, images are displayed on screens fixed very close in front of each eye. The screens are viewed through powerful convex lenses, so that the images appear to be located at a distance. A sense of depth is created by imposing a relative lateral offset of objects within the images presented to each eye, which in turn creates image disparity on the retina. The higher the lateral offset, the nearer the object appears. To prevent double vision as gaze shifts between objects, users make both version and vergence eye movements, which minimise retinal disparity of the object between eyes, and permit the object of interest to be perceived binocularly. In real world viewing of objects, vergence eye movements are associated with changes in accommodation to focus the eyes at the depth of the object. However, in VR, the focal distance of all objects on the screen is constant, and the eyes must converge without changing accommodation to maintain a clear retinal image. Thus, wearing a VR HMD creates a dissociation between convergence and accommodative demands^1^, which may contribute to visual discomfort^2–4^. Whether the effects of this disassociation persist after HMD use is unknown^5^, but a study investigating the effect of an early VR HMD system found a shift towards esophoria, and an extended near point of binocular convergence shortly after a 10-minute VR exposure^6^. Although VR technology has improved in recent years, the same optical limitations are present in current VR HMDs^7^.

Another potential issue which has received little attention is whether VR HMDs are likely to induce myopia (short-sightedness) with long term use. An association between near work and the development and progression of childhood myopia has long been recognised^8,9^. More specifically, the occupational use of microscopes has been linked with the development and progression of myopia in adults^10,11^, and microscopes and HMDs have similar near proximity cues: both involve binocular viewing of a close target through high powered lenses. In addition, recent research has implicated several other factors as important in myopia development and progression, including ambient light levels^12^, binocular status^13^, and refractive status of the peripheral retina^14^, all of which may be altered while using a VR HMD. Since the abnormal eye enlargement which underlies myopia development occurs over months and years, assessing the likely ‘myopiagenicity’ of a stimulus such as a VR HMD is challenging. However, the results of animal studies indicate that imposing hyperopic defocus on the retina with lenses (image plane located posterior to the retina), causes rapid thinning of the choroid, followed later by eye elongation and the development of myopia^15^. Conversely, creating myopic defocus (image plane located anterior to the retina), causes rapid thickening of the choroid, followed by a slowing of eye growth and the development of hyperopia^16^. Because of the predictable order in which these events occur, it is thought that the choroid is an intermediary between the retina and sclera, with changes in choroidal thickness reflecting changes in the eye-growth signalling pathway between retina and sclera^17^. Thus, an observed increase in choroidal thickness would indicate signals for reduced eye growth, whereas a decrease in thickness would indicate signals for accelerated growth and myopia development. Similar changes in the thickness of the human choroid have also been reported following short-term imposition of retinal defocus^18–21^ and it has been proposed that changes in choroidal thickness may serve as an indicator of pending changes to refractive status^18,22^. Therefore, monitoring the sign of any changes in choroidal thickness following a visual stimulus could provide a timely prediction of whether the stimulus would lead to myopia.

This study aimed to investigate whether wearing a current generation VR HMD altered binocular vision status, and whether it was likely to provide a myopiagenic stimulus if used over extended periods.

## Methods

### Study design

This was a prospective, multiple crossover study with four different environments in a two by two design: real and virtual indoor environments, and real and virtual outdoor environments. Each participant (n = 19) experienced all four environments in a randomised order. Participants were aged between 18–35 years (mean age 24.7 SD 4.0 years, 10 females), and were either emmetropic (n = 9) or wore their habitual contact lenses (n = 10, mean refractive error −1.66 SD 2.20D) to eliminate spectacle lens prism and difficulties fitting glasses under the HMD.

Participants gave written informed consent, were free to withdraw at any stage without giving reason, and their data has been deidentified and presented as pooled distributions. Exclusion criteria included stereopsis worse than 200 minarc, treatment for the progression of myopia (e.g. atropine, orthokeratology, or myopia control contact lenses), corrected visual acuity poorer than 6/7.5 in either eye, or a history of motion-sickness.

All participants took a battery of tests immediately prior, then immediately after exposure to each environment. The tests were completed in the following order: fixation stability, binocular vision tests and then choroidal thickness measures. After 40 minutes inside each environment, the battery of tests was repeated in reverse order: choroidal thickness, binocular vision, and then fixation stability, so that choroidal thickness was measured immediately before and after the trial. All tests were completed within five minutes of completing each environment, with equipment located to minimise participant movement. The optics of the HMD had a fixed lens centre separation of 65 mm, and light emitted from the headset focussed at approximately 1-meter from the headset. Participants were instructed to align the HMD on their head so that the screen appeared clear. A default stereoscopic camera setup was used with an in-game height of 180 cm, and a lateral camera separation of 65 mm. The participants were instructed on the use of the gamepad to move and interact in the virtual world, and they could converse with the experimenters if required. To normalise the conditions before each trial, participants worked at a computer for at least 45 minutes prior to baseline measurements. Each participant began each trial on different days, but at a similar time of day, to minimise any diurnal effects. The study was approved by The University of Auckland Human Participants Ethics Committee (Ref: 015908), and adhered to the Tenets of the Declaration of Helsinki.

### Environments

Each environment had a different combination of perceived viewing distance, accommodative demand, proximity cues for accommodation, convergence demand and luminance, to assist in determining which environmental parameters or combinations, might be important in any observed effects of HMD wear.

**Figure 1:**
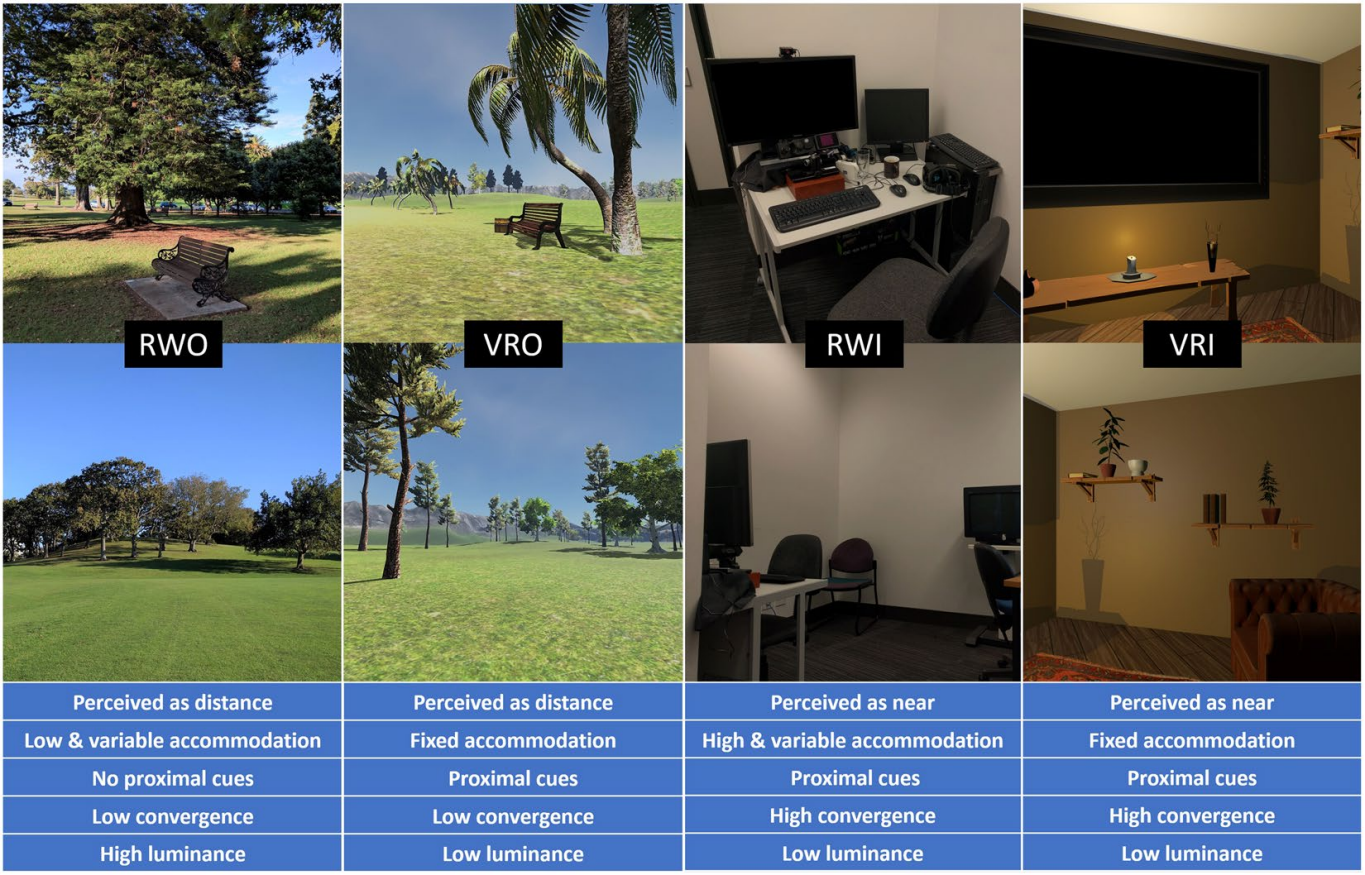
Examples of the four environments and comparison of their features. The outdoor real (RWO) and virtual (VRO) environments were brighter and spacious, while the indoor real (RWI) and virtual (VRI) environments were dimmer and more contained. Both virtual environments had constant accommodative demands, while accommodative demands in the real world varied by fixation distance. All environments except RWO had proximal near cues – in the virtual reality environments proximal cues were also due to the perception of wearing a headset.

The virtual environments were created in the Unity game engine (Version 5, Unity Technologies, USA), compiled for the Oculus DK2 (SDK version 0.8.0) and executed on a virtual reality capable machine (Intel Xeon E5–1650, nVidia GTX970, 16 Gb RAM). Audio was 3-D positional, and delivered through over-the-ear headphones to maximise immersion. The VR outdoor (VRO) environment consisted of a 1 km^2^ island, with features designed to mimic the real-world environment (Fig. 1). The island was bounded by water (to expose a distant horizon) on one side, and by mountain ranges along the three other sides. The island was scattered with ruins, hills, trees, and other items which rewarded exploration, and there were several treasure chests around the island which the participants were encouraged to find. Maximum speed of movement was restricted to a fast walking pace (1.5 meters per second) to minimise nausea, and to mimic the instructions given for the real-world environments. The VR indoor (VRI) environment was a small (~9 m^2^) cabin with dim lighting, and a large virtual television on the wall. The television played a documentary on the future of virtual reality (https://goo.gl/aDSGNr), and there were many objects on shelves and a table inside the cabin which provided a range of vergence demands. The maximum viewing distance in this environment was 3.5 m, designed to match the indoor environment in the real world. Light levels, measured by placing a lux meter sensor (LT300, Extech, USA) inside the headset in a darkened room, was approximately 180 lux in VRO, and 130 lux in VRI.

The real world outdoor (RWO) environment was the Auckland Domain, a large city park across the road from the University campus. Participants were instructed to walk around the park, avoiding near activities such as reading or using cell phones. Light level was measured during the middle of each RWO trial, and ranged from 150 to 90000 lux (median: 45000, interquartile range: 24500–81500 lux). The real world indoor (RWI) environment was a small office (approximately 9 m^2^), with no window. As was the case for the virtual indoor room, there was a range of items at various distances, and the maximum viewing distance was 3.5 m. Participants remained seated, and were encouraged to either work on or watch videos on an LCD computer monitor at approximately 1 m viewing distance. Overhead florescent tubes provided a 210-lux illumination in the vertical plane at eye height. Heart rate was measured with a wrist worn optical tracker (Charge HR, Fitbit, USA) throughout each trial, and served as a timer for the 40-minute trial duration.

### Binocular Vision Tests

**Fixation disparity** and stability at 50 cm was assessed on a binocular infrared eyetracker (Eyelink 1000, SR Research, Canada), which was calibrated before every measurement. The eyetracker camera was set to ‘remote’ mode, which uses a calibrated sticker positioned on the forehead to allow more naturalistic free-space measures, rather than requiring a chin/headrest. Participants were instructed to look at a maximum contrast target consisting of a combined bullseye and crosshair, which has been shown to be the most stable fixation target^23^. The target subtended 0.85 degrees, and was in the centre of an LCD monitor (Asus VG278H, 1920 × 1080 resolution, 120 Hz refresh, 28 pixels per degree). After a one second stabilisation period, the eyetracker recorded binocular eye position at 500 Hz for 4 seconds. If monitoring of the pupil or corneal reflex was disturbed during this period (e.g. due to a blink), the data was discarded and the test restarted. The pixel co-ordinates of the gaze position of each eye were captured directly into Matlab (2016a, Mathworks, USA). Fixation disparities were calculated as right eye minus left eye, with the result that a positive horizontal value represented an uncrossed ocular misalignment, and a positive vertical value a right eye hypo- misalignment.

**Fixation stability** was computed as 95% confidence interval bivariate contour ellipse areas (BCEA, minarc^2^) of binocular eye movements within the four second measurement interval^24,25^, as:

$$95 \% BCEA=2.291\times \pi \times {\sigma }_{x}\times {\sigma }_{y}\times \sqrt{1-{p}^{2}}$$

where σ_x_ = horizontal SD, σ_y_ = vertical SD, 2.291 is the χ^2^ value corresponding to two standard deviations, and *p* is the Pearson product moment correlation coefficient between X and Y data.

**Accommodation, phorias and stereopsis** Tests of binocular vision status included amplitude of accommodation (mean of three binocular push-up RAF rule measures), dissociated phorias at both distance (6 m) and near (0.4 m) using modified Thorington^26^, and stereopsis (Wirt Stereo fly test, Stereo Optical Co Inc). Inter-pupillary distance at both distance and near (0.4 m) were measured (Digital pupillometer, Essilor, France), so the effect of HMD lens decentration could be computed and correlated with changes in binocular vision, fixation stability, and fixation disparity.

**Choroidal thickness**, used as a proxy for the risk of myopia progression^22,27^, was measured using swept-source optical coherence tomography (SS-OCT, Atlantis DRI, Topcon, Japan) in horizontal and vertical cross scan mode (6 mm length, 1024 intervals, 96 averaged samples per line). The SS-OCT uses 1050 nm light to better penetrate the choroid, and minimises visibility of the scan line, which reduces patient tracking. The scan takes less than two seconds, and captures 100,000 A-scans per second at a resolution of 20 μm across the surface, and 8μm in depth, and is interpolated to give 1024 measures across the full 6 mm length. As the environmental exposure was binocular, but the eyes are not independent, one eye was randomly determined by coin-toss per-participant at the first visit, and this eye was used for all subsequent visits (42% left eyes). Automatic segmentation of the choroidal-scleral interface was performed by the machine software (Topcon FastMap, Version 9, Topcon Medical Systems, USA), and the segmented data was exported into Matlab for analysis. In rare cases where any of the 96 scan lines were missed (e.g. due to blink), or if the software reported poor choroidal image quality (<30), or if the choroidal-scleral boundary was incorrectly identified by the automated software, the scan was immediately repeated. Mean choroidal thickness measures, centred on the middle of the scan, were calculated for across the subfoveal (central 1 mm), parafoveal (3 mm) and perifoveal regions (6 mm)^28^.

### Statistical analysis

Baseline comparisons were normally distributed and compared with 1-way ANOVA with environment as the factor. Some of the changes (post-pre) in the measures of binocular vision, fixation stability, and choroidal thickness were not normally distributed, and were compared with non-parametric Kruskal Wallis tests, with post-hoc, paired-wise testing using Wilcoxon–Mann–Whitney using Šidák p-value correction for multiple comparisons, as required. As all 19 participants completed four environments, this gave 3 degrees of freedom between factors and 75 total degrees of freedom for all measures. Correlations were made with Pearson’s R. Heart rate was monitored throughout each trial, rather than pre and post, so the mean heart rate measure of the 40-minute period was compared between conditions. The datasets analysed during the current study are available from the corresponding author on reasonable request. Differences were treated as significant at p < 0.05.

## Results

### Binocular Vision

There were no significant differences in distance or near phoria, stereopsis, amplitude of accommodation, or fixation disparity between conditions at baseline (i.e. pre-trial, Table 1).

**Table 1:** Distribution of Binocular Vision (BV) parameters at baseline. There were no significant differences in any of the BV parameters prior to starting the trials: virtual reality indoor (VRI), virtual reality outdoor (VRO), real world indoor (RWI) and real world outdoor (RWO). MT = Modified Thorington, AoA = Amplitude of Accommodation, EsoP = Esophoria, ExoP = Exophoria.

	MT – 6 m	MT – 0.4 m	Stereopsis	AoA	Fixation Disparity
VRI	0.14EsoP ± 1.59^∆^	1.66ExoP ± 3.06^∆^	25 ± 8″	10.0 ± 1.6D	0.49^∆^ ± 0.35
VRO	0.20EsoP ± 1.55^∆^	1.79ExoP ± 2.46^∆^	23 ± 7″	9.7 ± 1.5D	0.45^∆^ ± 0.20
RWI	0.14EsoP ± 1.63^∆^	1.42ExoP ± 2.30^∆^	23 ± 4″	10.4 ± 1.7D	0.37^∆^ ± 30
RWO	0.33EsoP ± 1.89^∆^	1.29ExoP ± 2.10^∆^	27 ± 9″	9.8 ± 1.9D	0.37^∆^ ± 0.24
p	0.983	0.927	0.612	0.912	0.49

**Figure 2:**
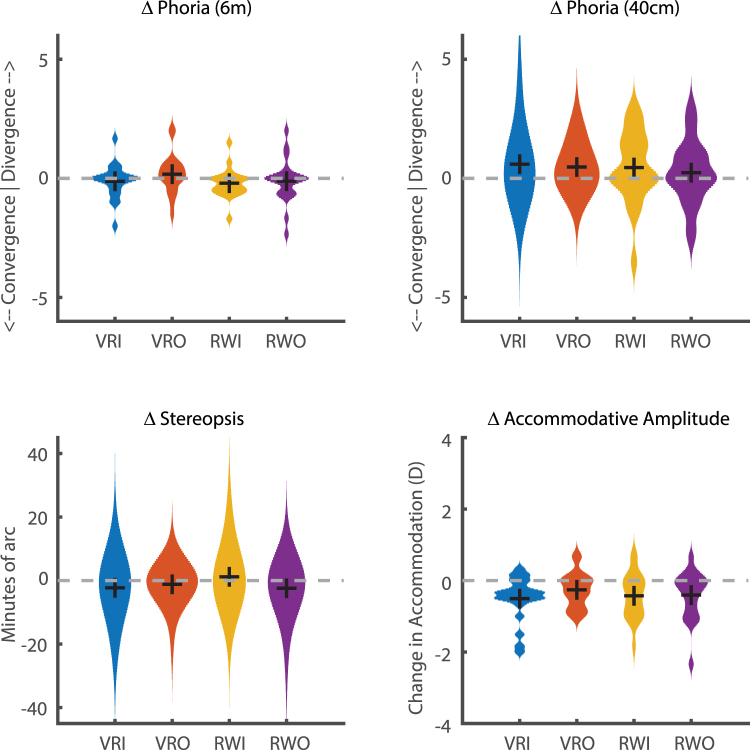
Change in binocular status after 40-minute exposure to virtual reality indoor (VRI), virtual reality outdoor (VRO), real world indoor (RWI) and real world outdoor (RWO) environments. There was no significant difference in the effect of environment on any of the four measures of binocular vision (Kruskal Wallis, all p > 0.05). Black crosses indicate group medians.

The change in binocular status (post-pre) was compared between each environment (Fig. 2). There was no significant difference in the change of the distance phoria (VRI: −0.22 SD 0.618∆, VRO: 0.23 SD 0.843∆, RWI: −0.30 SD 0.495∆, RWO: −0.13 SD 0.968∆, χ^Δ^ = 7.235, p = 0.065), near phoria (VRI: 0.66 SD 1.334∆, VRO: 0.53 SD 0.920∆, RWI: 0.55 SD 1.563∆, RWO: 0.26 SD 1.418∆, χ^2^ = 0.711, p = 0.871), maximum amplitude of accommodation (VRI: −0.54 SD 0.639D, VRO: −0.29 SD 0.505D, RWI: −0.44 SD 0.653D, RWO: −0.45 SD 0.694D, χ^2^ = 0.866, p = 0.834), or stereopsis (VRI: −2.5 SD 8.6″, VRO: −1.3 SD 4.7″, RWI: +1.6 SD 8.3″, RWO: −2.7 SD 7.1″, χ^2^ = 2.194, p = 0.533).

Participants had a mean inter-pupillary separation of 63.1 SD 2.58 mm (range 59–68 mm) at distance, and 58.8 SD 2.53 mm (range 55–64 mm) at near (0.4 m). The difference between an individual’s pupillary distance and the fixed lens separation distance in the HMD (65 mm) was computed as the lens centre offset: higher absolute values invoke greater horizontal prism as a factor of lens power. For participants with pupillary distances equal or less than the lens separation, this prism creates additional convergence demand during near viewing. Participants whose pupillary distance is greater than the lens separation, the induced prism depends on viewing distance: a greater divergence demand during distance viewing, and greater convergence during near viewing. Lens decentration was not correlated with individual changes in distance phoria (VRO: r = 0.412, p = 0.064, VRI: r = −0.142, p = 0.540) near phoria (VRO: r = 0.040, p = 0.864, VRI: r = −0.354, p = 0.115), or the change in maximum accommodation (VRO: r = −0.157, p = 0.497, VRI: r = −0.158, p = 0.494).

### Fixation Disparity

When the eyes converged on near objects in VR, the off-axis viewing through the powerful HMD lenses would have induced opposing prism to each eye. For the lens powers, viewing distances, and mean pupillary distances involved in this study, we calculate that this prismatic effect would approximately double the convergence demand compared to real world viewing of near objects, and thus could have doubled the potential stress to the visual system. In contrast, vertical off-axis viewing through the HMD lenses would have induced yoked prism, which would effectively cancel, and therefore require no adaptation. We separated fixation disparity into horizontal and vertical components to investigate the effect of any prismatic adaptation. There were no differences in baseline measures prior to environmental exposures for either horizontal (F = 0.43, p = 0.733), nor vertical fixation disparity (F = 0.4, p = 0.757). Also, there were no differences between the magnitudes of horizontal and vertical disparities (t_(75)_ = −1.051, p = 0.297).

Post-trial, there was no difference in the change of fixation disparity between environments in either the challenged horizontal direction (VRI: 0.08 SD 0.56∆, VRO: −0.13 SD 1.22∆, RWI: −0.01 SD 0.60∆, RWO: 0.06 SD 0.60∆, χ^2^ = 0.874, p = 0.832), nor the unchallenged vertical direction (VRI: 0.22 SD 0.54∆, VRO: 0.00 SD 0.63∆, RWI: −0.14 SD 0.51∆, RWO: 0.20 SD 0.38∆, χ^2^ = 6.709, p = 0.082). Although participants with smaller inter-pupillary distances would have experienced higher prismatic effects, there was no correlation between an individual’s change in horizontal fixation disparity and their lens decentration in either VRI (r = 0.224, p = 0.330) or VRO (r = −0.28, p = 0.219) groups.

### Fixation Stability

Fixation stability, defined as a bivariate contour ellipsoid area (BCEA) containing 95% of individual fixation samples, was consistent at baseline (VRI: 0.53 SD 0.406 minarc^2^, VRO: 0.80 SD 0.513 minarc^2^, RWI: 0.68 SD 0.392 minarc^2^, RWO: 0.67 SD 0.520 minarc^2^, F = 1.13, p = 0.343). BCEA did not change post-trial, and there was no obvious effect of virtual reality (VRI: 0.15 SD 0.450 minarc^2^, VRO: −0.06 SD 0.981 minarc^2^, RWI: 0.55 SD 1.229 minarc^2^, RWO: 0.61 SD 1.511 minarc^2^, χ^2^ = 1.57, p = 0.203).

### Choroidal Thickness

**Figure 3:**
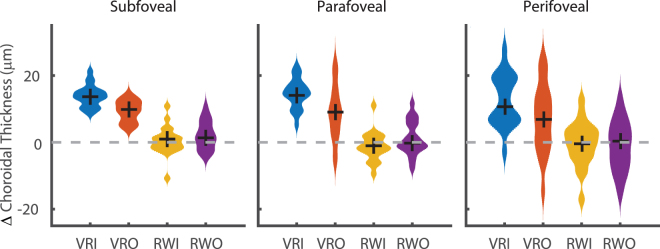
There was significant increase in the choroidal thickness after 40 minutes in both VR groups at subfoveal (1 mm) and parafoveal (3 mm) regions of the choroid (Kruskal Wallis, <0.001). In contrast, there was no significant increase in choroidal thickness in either of the real-world groups. Black crosses mark group medians.

There was no difference in the baseline choroidal thickness values before starting each environment (subfoveal: Mean: 294.1 SD 95.4 µm, F = 0.08, p = 0.971, parafoveal: Mean: 292.8 SD 89.3 µm, F = 0.06, p = 0.981, perifoveal: Mean: 280.0 SD 78.2 µm, F = 0.08, p = 0.973). After the exposure, there was a significant difference in the choroidal thickness change between the different environments in the subfoveal (VRI: +13.9 SD 3.3 µm, VRO: + 9.3 SD 3.2 µm, RWI: + 0.5 SD 4.4 µm, RWO: +2.1 SD 4.3 µm, χ^2^ = 51.7, p < 0.001, Fig. 3) and parafoveal zones (+14.6 SD 4.5 µm, VRO: +9.0 SD 8.3 µm, RWI: −1.2 SD 4.4 µm, RWO: +1.2 SD 5.1 µm, χ^2^ = 42.6, p < 0.001). The subfoveal increase after VRI was larger than both RW groups (both p < 0.001), as was the change following VRO (vs RWI p < 0.001, RWO p = 0.003). The subfoveal change in choroidal thickness was not different between VRO and VRI (p = 0.174). In the parafoveal area, the change in VRI was greater than both real-world groups (both p < 0.001), as was the change in choroidal thickness after VRO (vs RWI: p < 0.001, RWO: p = 0.025). Over the full 6 mm perifoveal scan length, despite higher variance, there was still a difference between groups (VRI: 12.5 SD 7.8 µm, VRO: 7.2 SD 10.4 µm, RWI: −0.4 SD 6.8 µm, RWO: −1.0 SD 7.6 µm, χ^2^ = 25.5, p < 0.001). VRI was significantly higher than both RWI and RWO (both p < 0.001) groups, but VRO was not (RWI, p = 0.085, RWO, p = 0.059). Individual changes in choroidal thickness were not correlated with light levels (r − 0.02, p = 0.149), nor lens decentration (r = 0.04 p = 0.563).

### Heart Rate

**Figure 4:**
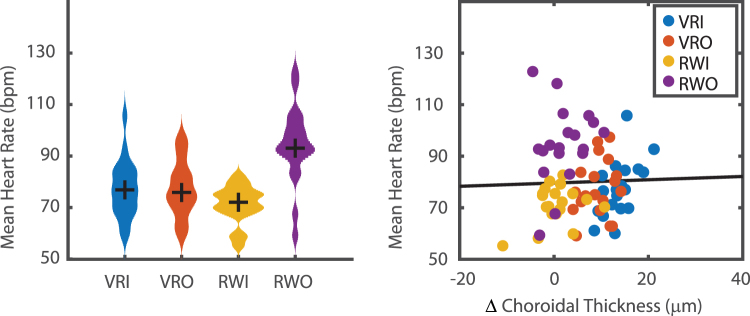
Heart rate was significantly higher in RWO (left figure, Kruskal Wallis, p < 0.001), but was not different between RWI, VRI, or VRO. However, the mean heart rate during each trial was not correlated in the change in choroidal thickness (right, Pearson’s r = 0.03, p = 0.800).

Heart rate was continuously measured during each 40-minute environmental exposure. Participants had a higher heart rate (beats per minute, bpm) during the VRO condition than all other conditions (VRI: 77.3 SD 11.1 bpm, VRO: 77.3 SD 10.9 bpm, RWI: 71.2 SD 7.4 bpm, RWO: 94.2 SD 14.0, χ^2^ = 24.7, p < 0.001, Fig. 4 left). There was no difference in heart rate between the two VR conditions (p = 0.999), nor VRI and RWI (p = 0.545), nor VRO and RWI (p = 0.600). There was also no correlation between heart rate and the change in choroidal thickness (n = 76, r = 0.03, p = 0.800, Fig. 4 right).

## Discussion

A 40-minute exposure to a VR HMD environment appears to have minimal effects on the binocular vision system. The fixed accommodative demand in a HMD, and the associated accommodative-convergence disconnect did not affect the dissociated position of the eyes either at distance or near, nor the maximum amplitude of accommodation, suggesting no accommodative fatigue. As the HMD lenses are relatively high powered (approximately 25D), eye movements away from the lens centres will have induced large prismatic effects. While this is of little importance for version eye movements where the prism will be yoked, vergence eye movements, stimulated by viewing objects at different depths, will create asymmetric prism and potentially binocular vision stress. If eye position (and therefore induced prism) remains relatively constant, such as the case in the indoor VR scene, the constant prism could cause the binocular vision system to adapt (which would then be expected to re-adapt once out of VR). However, we found no difference in binocular fixation disparity between horizontal and vertical directions, and all measures fell well within reported normal population ranges^25^. This indicates that if there is any binocular stress, it is likely minimal or able to dissipate by the time second measurement was taken (between five and ten minutes post exposure), and is not significantly higher after VR use compared to an equivalent task conducted in the real world.

While there were no overall effects of VR on the binocular vision system in our adult cohort, there may still be of concerns if children use VR headsets which have been calibrated for adults, as children tend to have smaller pupillary distances^29^. Some VR headsets allow the lens centration distance to be adjusted, but many phone-based headsets, which may be more available to children, do not currently allow lens separation adjustment.

Somewhat unexpectedly, we found a significant increase in choroidal thickness after VR HMD wear. As briefly discussed in the introduction, the opposite effect (choroidal thinning) would be expected to occur when the eye is exposed to a myopiagenic stimulus^17^. This effect may be caused by the unique optical arrangement inside HMDs causing a lead of accommodation. Choroidal thickening occurs after myopic defocus^20,30^, which is associated with a slowing of eye growth^16^, and a reduction in myopia progression^31^. We hypothesise that the choroidal thickening effect seen after VR HMD wear is a consequence of the fixed viewing distance, combined with convergence-induced accommodation in the virtual environment. Viewing objects at near in the real world involves both accommodation and convergence, but is typically associated with a *lag* of accommodation^32^, and therefore hyperopic retinal defocus, which has been proposed as a stimulus for myopia development and progression^33^. However, the lenses in a HMD are generally set for distance viewing (i.e. primary position of gaze), and convergence of the eyes results in off-centre viewing through the lenses. This induces a base-out prismatic effect, and an image displacement which increases the amount of convergence required compared to an equivalent real-world task. Thus, participants would need to execute exaggerated convergent eye movements to view virtual near objects. This would be expected to strengthen any associated convergence-induced accommodation^34^, and as the HMD screen distance is fixed, this would create a *lead* of accommodation and a myopically defocussed retinal image, which causes choroidal thickening^20^. Relatively small amounts of image defocus are not necessarily associated with a decrease in visual acuity^35^, and the limited spatial resolution of the screens would help mask any subtle defocus. The more contained indoor virtual environment would have been associated with higher convergence, thus more convergence-induced accommodation and greater myopic retinal defocus compared to the distant virtual outdoor environment. Therefore, the virtual indoor environment would be expected to show greater choroidal thickening than the virtual outdoor environment, which our results support. However, further longitudinal research is required to determine whether this change in choroidal thickness could influence myopia progression. Future improvements to VR displays, such as the move towards light field displays, will address the convergence-accommodative conflict, but would also remove this potential lead of accommodation as a protective mechanism against myopia. Another factor which may have caused the eyes to accommodate and thus created myopic retinal defocus while wearing the HMD, is the effect of proximal accommodation^36^, and this may help account for the observation that both VR environments were associated with choroidal thickening, while the real environments were not. An alternative explanation for choroidal thickness could be changes in ambient air temperature within the HMD, or modification of blood flow directly related to the wearing of the headset itself. The choroid is a dense vascular layer at the back of the eye, which also has a function to draw heat energy away from the eye^37,38^, and changes in peripheral blood vessel dilation can be detected through changes in ocular surface temperature^39^. While wearing the VR headset, the air temperature inside the headset quickly approached skin temperature, and this may have influenced the thermal gradient from the anterior to posterior eye by modifying tear film evaporation^40^ or aqueous humour dynamics^41^, ultimately causing a change in choroidal blood flow. It should be noted that our preconditioning — in which all participants used a computer for at least 45 minutes — would have likely caused thinning of the choroid at baseline, and this is supported by the minimal change in choroidal thickness after the real world indoor condition. Therefore, we can only say that VR caused choroidal thickening relative to an equivalent real world task.

While the study was well powered using a repeated measures cross-over design, there are limitations to the extrapolation of our results. The 40 minute trial length was a compromise between convenience for participants, while being sufficiently long enough to detect changes in choroidal thickness^20^, and much longer than previous studies^6^ which were able to show an effect on binocular vision. However, in practice, some users are likely to spend much longer periods than 40 minutes in VR. Also, our study only investigated the effect on a cohort with normal binocular vision. As converging on near objects in VR creates a higher convergence demand than in real world viewing, those with abnormal binocular vision, such as myopic children who may already excessively converge at near^42^, could still become symptomatic. Further, while the choroidal thickness change is promising, we used an adult cohort who are unlikely to be at significant risk of myopic progression. Additional work is needed to investigate the cause of this change, and whether the mechanism of choroidal thickening could help protect against myopia progression in children.

## Conclusions

Using a VR headset for 40-minutes did not appear to affect the binocular vision status, compared to a real world equivalent task. However, an unexpected finding was that choroidal thickness markedly increased when using a VR headset. We hypothesise that this was due to convergence-induced accommodation when viewing near virtual objects which would have created a myopically defocussed retinal image of the virtual environment because of the fixed viewing distance. Myopic retinal defocus causes choroidal thickening in humans: it is also associated with a reduced myopia progression rate in children. On this basis, our results suggest that VR HMD wear may not provide a myopia-inducing stimulus despite the close viewing distances involved.

## References

[1] Vienne C, Sorin L, Blondé L, Huynh-Thu Q, Mamassian P (2014). Effect of the accommodation-vergence conflict on vergence eye movements. Vision Res..

[2] Shibata T, Kim J, Hoffman DM, Banks MS (2011). The zone of comfort: Predicting visual discomfort with stereo displays. J. Vis..

[3] Lambooij M, IJsselsteijn W, Fortuin M, Heynderickx I (2009). Visual Discomfort and Visual Fatigue of Stereoscopic Displays: A Review. J. Imaging Sci. Technol..

[4] Howarth PA (2011). Potential hazards of viewing 3-D stereoscopic television, cinema and computer games: a review. Ophthalmic Physiol. Opt..

[5] Rushton SK, Riddell PM (1999). Developing visual systems and exposure to virtual reality and stereo displays: some concerns and speculations about the demands on accommodation and vergence. Appl. Ergon..

[6] Mon-Williams M, Wann JP, Rushton S (1993). Binocular vision in a virtual world: visual deficits following the wearing of a head-mounted display. Ophthalmic Physiol. Opt..

[7] Wann JP, Rushton S, Mon-Williams M (1995). Natural problems for stereoscopic depth perception in virtual environments. Vision Res..

[8] Mutti DO, Mitchell GL, Moeschberger ML, Jones LA, Zadnik K (2002). Parental myopia, near work, school achievement, and children’s refractive error. Investig. Ophthalmol. Vis. Sci..

[9] Hepsen IF, Evereklioglu C, Bayramlar H (2001). The effect of reading and near-work on the development of myopia in emmetropic boys: a prospective, controlled, three-year follow-up study. Vision Res..

[10] McBrien NA, Adams DW (1997). A longitudinal investigation of adult-onset and adult-progression of myopia in an occupational group. Refractive and biometric findings. Invest. Ophthalmol. Vis. Sci..

[11] Ting PWK, Lam CSY, Edwards MH, Schmid KL (2004). Prevalence of Myopia in a Group of Hong Kong Microscopists. Optom. Vis. Sci..

[12] Norton TT, Siegwart JT (2013). Light levels, refractive development, and myopia - A speculative review. Exp. Eye Res..

[13] Anderson H, Stuebing KK, Fern KD, Manny RE (2011). Ten-Year Changes in Fusional Vergence, Phoria, and Nearpoint of Convergence in Myopic Children. Optom. Vis. Sci..

[14] Smith EL (2013). Optical treatment strategies to slow myopia progression: Effects of the visual extent of the optical treatment zone. Exp. Eye Res..

[15] Wildsoet C, Wallman J (1995). Choroidal and scleral mechanisms of compensation for spectacle lenses in chicks. Vision Res..

[16] Wallman J (1995). Moving the retina: Choroidal modulation of refractive state. Vision Res..

[17] Nickla DL, Wallman J (2010). The multifunctional choroid. Progress in Retinal and Eye Research.

[18] Wang, D. *et al*. Optical defocus rapidly changes choroidal thickness in schoolchildren. *PLoS One* **11** (2016). 10.1371/journal.pone.0161535 PMC4990278 27537606

[19] Chakraborty R, Read SA, Collins MJ (2012). Monocular myopic defocus and daily changes in axial length and choroidal thickness of human eyes. Exp. Eye Res..

[20] Chiang ST-H, Phillips JR, Backhouse S (2015). Effect of retinal image defocus on the thickness of the human choroid. Ophthalmic Physiol. Opt..

[21] Read SA, Collins MJ, Sander BP (2010). Human optical axial length and defocus. Investig. Ophthalmol. Vis. Sci..

[22] Read SA, Alonso-Caneiro D, Vincent SJ, Collins MJ (2015). Longitudinal changes in choroidal thickness and eye growth in childhood. Investig. Ophthalmol. Vis. Sci..

[23] Thaler L, Schütz AC, Goodale MA, Gegenfurtner KR (2013). What is the best fixation target? The effect of target shape on stability of fixational eye movements. Vision Res..

[24] Bellmann C, Feely M, Crossland MD, Kabanarou SA, Rubin GS (2004). Fixation stability using central and pericentral fixation targets in patients with age-related macular degeneration. Ophthalmology.

[25] Morales MU (2016). Reference Clinical Database for Fixation Stability Metrics in Normal Subjects Measured with the MAIA Microperimeter. Transl. Vis. Sci. Technol..

[26] Cebrian JL (2014). Repeatability of the Modified Thorington Card Used to Measure Far Heterophoria. Optom. Vis. Sci..

[27] Fitzgerald MEC, Wildsoet CF, Reiner A (2002). Temporal Relationship of Choroidal Blood Flow and Thickness Changes during Recovery from Form Deprivation Myopia in Chicks. Exp. Eye Res..

[28] Read SA, Collins MJ, Vincent SJ, Alonso-Caneiro D (2013). Choroidal thickness in myopic and nonmyopic children assessed with enhanced depth imaging optical coherence tomography. Invest. Ophthalmol. Vis. Sci..

[29] MacLachlan C, Howland HC (2002). Normal values and standard deviations for pupil diameter and interpupillary distance in subjects aged 1 month to 19 years. Ophthalmic Physiol. Opt..

[30] Woodman EC, Read SA, Collins MJ (2012). Axial length and choroidal thickness changes accompanying prolonged accommodation in myopes and emmetropes. Vision Res..

[31] Phillips JR (2005). Monovision slows juvenile myopia progression unilaterally. Br. J. Ophthalmol..

[32] Charman WN (1999). Near vision, lags of accommodation and myopia. Ophthalmic Physiol. Opt..

[33] Gwiazda JE (2011). Progressive-addition lenses versus single-vision lenses for slowing progression of myopia in children with high accommodative lag and near esophoria. Investig. Ophthalmol. Vis. Sci..

[34] Schor C (1999). The influence of interactions between accommodation and convergence on the lag of accommodation. Opthalmology Physiol. Opt..

[35] Buehren T, Collins MJ (2006). Accommodation stimulus-response function and retinal image quality. Vision Res..

[36] Kotulak JC, Morse SE, Wiley RW (1994). The effect of knowledge of object distance on accommodation during instrument viewing. Perception.

[37] Parver LM (1991). Temperature modulating action of choroidal blood flow. Eye (Lond)..

[38] Parver LM, Auker C, Carpenter DO (1980). Choroidal blood flow as a heat dissipating mechanism in the macula. Am. J. Ophthalmol..

[39] Vannetti F (2014). Relationship between ocular surface temperature and peripheral vasoconstriction in healthy subjects: a thermographic study. Proc. Inst. Mech. Eng. H..

[40] Abusharha, A. A., Pearce, E. I. & Fagehi, R. Effect of Ambient Temperature on the Human Tear Film. *Eye Contact Lens* **42** (2016). 10.1097/ICL.0000000000000210 26595213

[41] Ooi EH, Ng EYK (2008). Simulation of aqueous humor hydrodynamics in human eye heat transfer. Comput. Biol. Med..

[42] Gwiazda J, Grice K, Thorn F (1999). Response AC/A ratios are elevated in myopic children. Ophthalmic Physiol. Opt..

